# Bacterial conjugation in the ruminant pathogen *Mycoplasma agalactiae* is influenced by eukaryotic host factors

**DOI:** 10.1128/aem.00868-25

**Published:** 2025-05-27

**Authors:** M'hamed Derriche, Laurent Xavier Nouvel, Maria Gaudino, Eveline Sagné, Elisa Simon, Hortensia Robert, Gwendoline Pot, Gilles Meyer, Christian de la Fe, Yonathan Arfi, Renaud Maillard, Christine Citti, Eric Baranowski

**Affiliations:** 1 IHAP, Université de Toulouse, INRAE, ENVT 54891 , Toulouse, France; 2 Ruminant Health Research Group, Departamento de Sanidad Animal, Facultad de Veterinaria, Universidad de Murcia 571776 , Murcia, Spain; 3 University of Bordeaux, INRAE, UMR BFP 27086, Villenave-d'Ornon, France; INRS Armand-Frappier Sante Biotechnologie Research Centre, Laval, Quebec, Canada

**Keywords:** bacterial conjugation, mycoplasma, mobile elements, integrative and conjugative elements, chromosomal transfer, epithelial cells, organotypic cultures

## Abstract

Horizontal gene transfer (HGT) plays a pivotal role in the evolution and adaptation of genome-reduced mycoplasmas. The conjugative properties of these organisms are key in this phenomenon but are largely understudied, particularly *in vivo*. In the present study, the ruminant pathogen *Mycoplasma agalactiae* was used as a model organism to document mycoplasma conjugation in environments of increasing complexity, from axenic to cell and organotypic culture conditions. Compared to axenic mating conditions, mycoplasma co-cultivation with goat epithelial cells or bovine precision-cut lung slices resulted in enhanced mating frequencies with high rates of *M. agalactiae* integrative and conjugative element (ICEA) self-dissemination. These results were conditioned by the presence of eukaryotic cells in the culture and influenced by competition between mating partners but were not limited to *M. agalactiae*, as similar results were observed with *Mycoplasma bovis*. Mycoplasma conjugation *ex vivo* was further characterized by analyzing mycoplasma chromosomal transfer (MCT), a newly discovered mechanism of horizontal exchange of chromosomal DNA that generates mosaic genomes. Compared to ICEA transfer, MCT was detected at lower rates under cell and organotypic culture conditions, suggesting a negative impact of these cellular environments on MCT or its progeny. Finally, mating experiments under nutrient-deprived conditions identified nucleotide stress as a potential factor influencing the modulation of mycoplasma conjugation by eukaryotic host cells. In conclusion, these results suggest that HGT *in vivo* is likely underestimated and provide valuable models to further study mycoplasma conjugation *ex vivo*.

**IMPORTANCE:**

Conjugation is an evolutionary shortcut that bacteria use to exchange genetic information with their neighbors. Despite the fast evolution rate of the genome-reduced mycoplasmas, their conjugative properties remain largely understudied, particularly *in vivo*. Here we used the ruminant pathogen *Mycoplasma agalactiae* to study how mycoplasmas conjugate in co-culture with host-derived cells and tissues. Interestingly, conjugation was stimulated when mycoplasmas were co-cultured with eukaryotic cells. This was documented by monitoring the self-propagation of a mobile genetic element known as integrative and conjugative element (ICE) and the exchange of chromosomal DNA leading to the formation of mosaic genomes. While ICE transfer was observed at high frequency, only a few mosaic genomes were detected in the presence of eukaryotic cells. Further data point toward nucleotide stress as a possible factor modulating mycoplasma conjugation in cellular environments. These results suggest that mycoplasma-host interactions may stimulate conjugation *in vivo*.

## INTRODUCTION

Bacterial conjugation is the predominant mechanism of horizontal gene transfer (HGT) in microbial communities. By enabling DNA to be transferred between two bacterial cells through direct contact, conjugation facilitates the acquisition and dissemination of new adaptive traits, including antimicrobial resistance (1–3). This horizontal process is classically mediated by plasmids or integrative and conjugative elements (ICE), which encode the conjugative machinery that promotes mating pair formation between donor and recipient cells and DNA transfer across a mating channel (4–7). Conjugation has been documented in a broad number of bacterial species, including mycoplasmas (8).

Mycoplasmas are fast-evolving, wall-less bacteria of the class *Mollicutes* that are restricted to an obligate parasitic lifestyle due to their reduced genomes (9–11). Members of the *Mycoplasma* genus have a predilection for mucosal surfaces of the respiratory and genital tracts of their hosts, with some species causing chronic, often debilitating infections in humans and animals (9, 10, 12). The control of pathogenic species is challenging, in particular due to alarming rates of antimicrobial resistance (10, 13, 14). Recently, a new mechanism of HGT known as mycoplasma chromosomal transfer (MCT) has been characterized in *Mycoplasma agalactiae*, a pathogenic ruminant *Mycoplasma* sp. (8, 15). MCT is an unconventional process that generates highly heterogeneous populations of mosaic genomes through the massive exchange of chromosomal DNA between mating partners (15–17). Following MCT, DNA fragments originating from any part of the donor genome replace regions of a few nucleotides to tens of kilobase pairs of the recipient chromosome at homologous sites (8, 15–17). Mainly documented *in vitro*, cumulative evidence suggests that MCT may also occur *in vivo* (18, 19).

**Fig 1:**
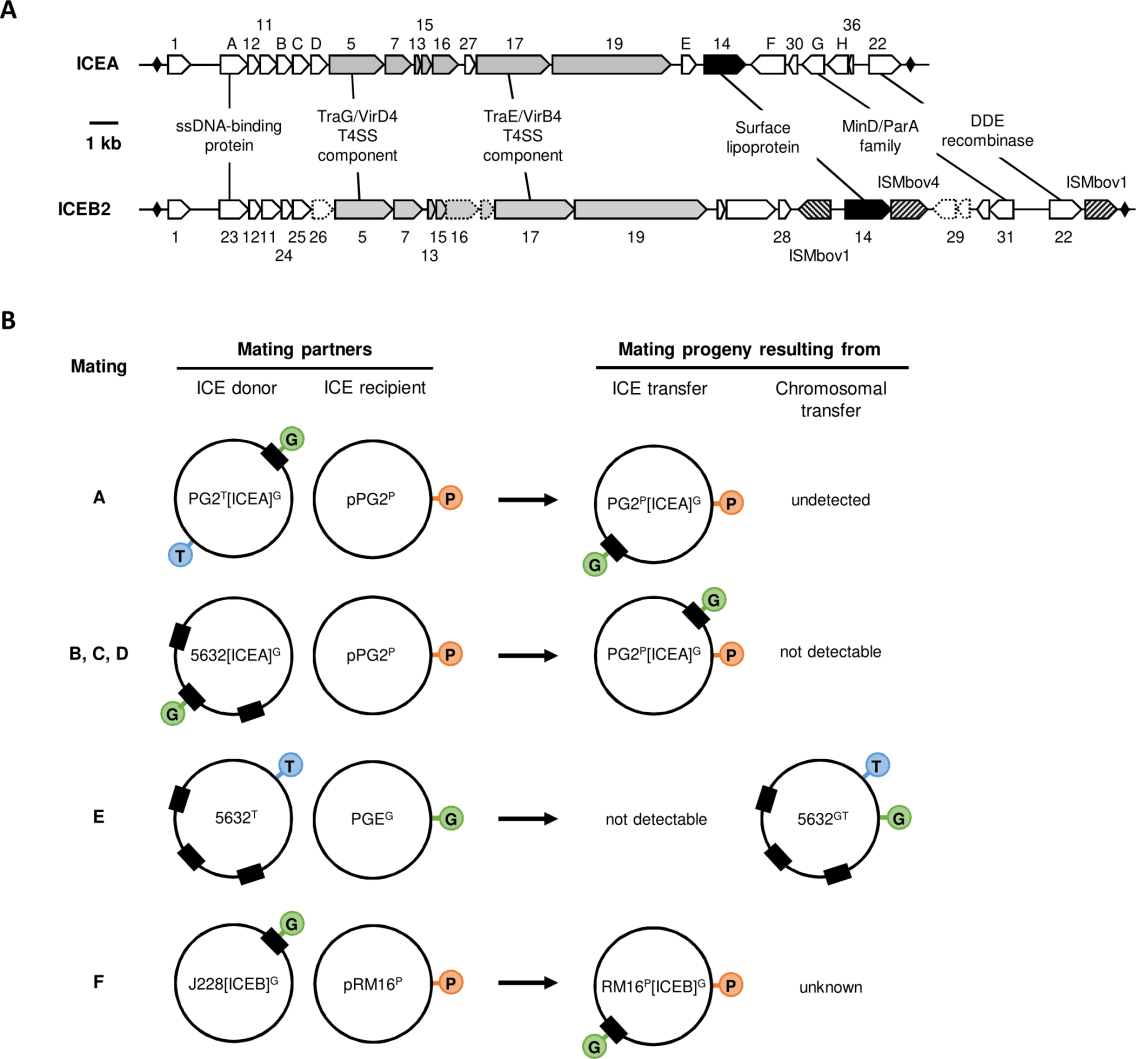
Integrative and conjugative elements and mating experiments with *M. agalactiae* and *M. bovis*. (**A**) Comparison of *M. agalactiae* ICEA and *M. bovis* ICEB2. The 27 kb ICEA and the 31 kb ICEB2 are shown with their respective coding sequences (CDSs). The two inverted repeats flanking ICEA and ICB2 are represented by black diamonds. The genes encoding predicted surface lipoproteins and those encoding proteins with putative transmembrane domains are shown in black and gray, respectively. Pseudogenes are indicated by dotted lines. Insertion sequences (IS) are indicated by hatched arrows. IS located on ICEB flanks were not included in the size calculation. Hypothetical CDS functions shared by ICEA and ICEB2 are indicated. (**B**) Illustration of the different mating experiments performed in the present study. For each mating experiment (indicated with a letter), the two partners are illustrated with their specific antibiotic markers (Ab-Tag) represented by colored circles. The ICEA G-tag is in green, and the chromosomal T- and P-tags are in blue and orange, respectively. The chromosomal ICEA is represented by a black box. The mating progenies resulting from ICEA or chromosomal transfer (CT) are illustrated, except when no progeny was detected (undetected), when the progeny could not be detected due to the absence of an appropriate Ab-tag (not detectable), or when not previously described (unknown). During ICE transfer, the Ab-tagged ICE is self-transferred from the donor chromosome to the Ab-labeled recipient chromosome, resulting in the generation of a dual-resistant progeny (20). CT has been extensively studied and shown to be independent from ICE transfer, yet it requires a functional ICE in one of the partners for conjugation. CT creates mosaic genomes by simultaneously replacing multiple chromosomal DNA regions from one partner to the other at homologous loci. In mating E, CT occurs only from PG2 to 5632 (15).

MCT is tightly coupled to conjugation, which is mediated by a self-transmissible ICE (8, 15, 20). Widely distributed across *Mollicutes*, mycoplasma ICEs are key mediators of horizontal gene flow in these organisms (8, 21). The best characterized of these self-transmissible elements is the functional ICE of *M. agalactiae* (ICEA; Fig. 1A) (20, 22). Unlike conventional ICEs which rely on site-specific tyrosine recombinases for their mobility, ICEA movements are mediated by a DDE transposase responsible for random, chromosomal integration events that generate a diverse population of transconjugants (20).

The *in vitro* study of mycoplasma HGT has been facilitated by the establishment of optimal mating conditions for ruminant mycoplasmas, including the use of axenic, enriched growth media that meet the nutritional requirements of these fastidious organisms (20, 23). However, functional genomics studies have shown that the biology of these organisms can be significantly influenced by their replicative environment, particularly when co-cultured with eukaryotic host cells (24–26). This raises questions about the influence of mycoplasma-host interactions on ICE-mediated conjugation and MCT. To address this issue, mating experiments were carried out with the model species *M. agalactiae* under replicative environments of increasing complexity, from *in vitro* to *ex vivo*, aiming to more closely mimic the *in vivo* conditions. When compared to optimal axenic conditions, mating experiments revealed enhanced conjugative activity upon mycoplasma co-incubation with eukaryotic host cells or precision-cut lung slices (PCLS) with important differences among strains. The dependence of mycoplasmas on host cells for nutrient acquisition prompted us to investigate the influence of nutritional stress on the mating process. Mating experiments under nutrient-deprived conditions identified nucleotides as a potential factor influencing mycoplasma conjugation under cell culture conditions.

## RESULTS

### Mycoplasma co-cultivation with epithelial cells induces high-frequency ICEA transfer

To date, the conjugative properties of *M. agalactiae* have only been studied in axenic conditions (8). Under these conditions, ICE donor and recipient cells are co-incubated in SP4 medium after centrifugation to promote contact between the mating partners (20, 23). Since these settings differ greatly from natural growth conditions, co-cultivation of *M. agalactiae* with host epithelial cells was used to assess mycoplasma conjugative capacity *in vivo*. Mating experiments were conducted with strain PG2, and antibiotic resistance markers (Ab-tags) were used to monitor ICEA transfer from donor to recipient cells (Fig. 1, mating A). The PG2^T^[ICEA]^G^ clone was used as the ICEA donor (Table 1). Its genome is characterized by the presence of (i) a functional ICEA tagged with a gentamicin (G) marker (G-tag) to follow ICE movements and (ii) a chromosomal tetracycline (T) marker (T-tag) to monitor chromosomal DNA exchange between mating partners (see below). The ICEA recipient partner consisted of a pool of five puromycin (P)-resistant PG2 clones (further designated pPG2^P^) that was used to minimize any negative effect resulting from the chromosomal insertion of the P-tag (Table 1).

**TABLE 1:** Ab-tagged mycoplasma strains used in mating experiments

Ab-tagged strains	Comments
*M. agalactiae^a^*	
PG2^G^	ICEA-negative, G-tagged PG2 mutant T8.101 (24)
pPG2^P^	Pool of five ICEA-negative PG2 mutants, each having a chromosomal P-tag inserted randomly by transformation with pMT85Pur
PG2^T^[ICEA]^G^	ICEA-positive PG2^T^[ICEA *ncr19/E::mTn*]^G^ clone 23 (22). This clone is a T-tagged PG2, which has acquired a G-tagged ICEA from 5632[ICEA]^G^ upon mating
5632^T^	ICEA-positive 5632 clone H3 having a chromosomal T-tag inserted outside the three ICEA regions (15)
5632[ICEA]^G^	ICEA-positive 5632[ICEA *ncr19/E::mTn*]^G^ clone 23 having a G-tag inserted within ICEA non-coding region 19/E (intergenic region between CDS19 and CDSE) with no influence on conjugation (22)
*M. bovis^b^*	
pRM16^P^	Pool of five ICEB2-negative RM16 mutants, each having a chromosomal P2-tag inserted by transformation with pMT85Pur2
J228[ICEB]^G^	ICEB2-positive J228[ICEB2 *ISMbov1*::*mTn*]^G^ clone 277 having a G-tag inserted within ICEB2 *ISMbov1* located between ICEB2 CDS28 and 14

^*a*^ *M. agalactiae* strains PG2 and 5632 have been previously described (19, 27). Their genome sequences are available under GenBank accession numbers CU179680.1 and FP671138.1.

^*b*^ *M. bovis* strains RM16 and J228 have been previously described (25, 28). Their genome sequences are available under GenBank accession numbers CP077758.1 and CP068731.1.

**Fig 2:**
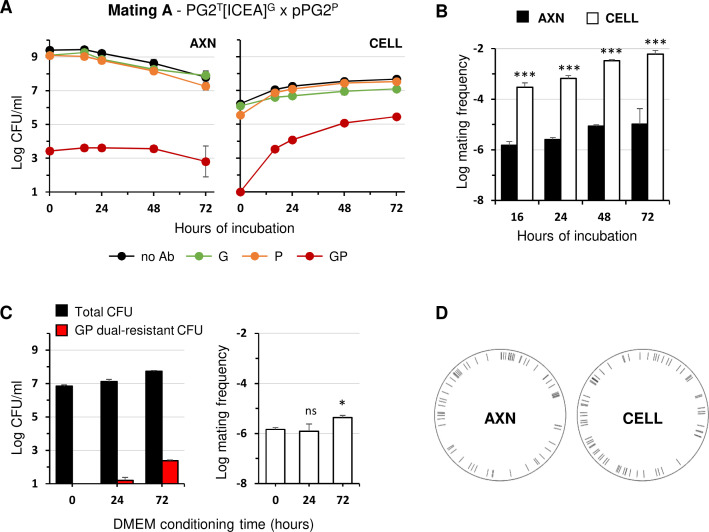
Mating experiments with *M. agalactiae* strain PG2 under axenic (AXN) and cell culture (CELL) conditions. (**A**) Variations in mycoplasma titers under AXN and CELL mating conditions with ICEA-positive and ICEA-negative PG2 mating partners (mating A in Fig. 1). Ab-tagged partners and their dual-resistant offspring were analyzed by plating on selective media. Antibiotics used for selection are color-coded (green for G, orange for P, red for G and P). Mycoplasma titers without Ab selection are shown in black. (**B**) Mating frequencies under AXN and CELL conditions at different time points. Mating frequencies were calculated as the number of dual-resistant transconjugants per total CFU. (**C**) Mycoplasma titers and mating frequencies after 24 h of incubation in conditioned Dulbecco’s modified Eagle’s medium (DMEM) with ICEA-positive and ICEA-negative PG2 mating partners (mating A in Fig. 1B). The conditioned DMEM was pre-cultured (0, 24, or 72 h) with the epithelial cells (1.5 × 105 cells/mL). Cell debris was removed from the conditioned DMEM supernatant by centrifugation at 5,000 *× g* and 0.2 µM filtration. (**D**) Distribution of unique chromosomal ICE-integration sites identified in AXN and CELL mating progeny. Data are means of at least three independent experiments. Standard deviations are indicated by error bars. *P*-values were determined using two-tailed *t*-tests for independent samples comparing the mating frequency under cell culture and axenic conditions at different incubation times (ns, *P* > 0.05; *, *P* < 0.05; ***, *P* < 0.001).

After increasing incubation times under previously defined axenic or cell culture mating conditions, Ab-tagged partners and their dual-resistant progeny were counted by plating on selective media (Fig. 2A). Under axenic conditions, a progressive decline in mycoplasma titers of the parent strains was observed, which is attributable to the high initial cell density of the inoculum (10^9^ CFU/mL) classically used in this assay. Dual-resistant colonies were detected right after mixing the mating partners under axenic conditions (see 0 h of incubation in Fig. 2A). These are likely resulting from mating events that occurred shortly after the centrifugation step used to promote contact between mycoplasma cells. In contrast, the cell culture assay was conducted with low initial density of mating partners (10^6^ CFU/mL), as initially designed in previous studies (24, 29). As expected, this low initial load resulted in bacterial growth (Fig. 2A).

Both mating conditions generated dual-resistant colonies characterized by gentamicin and puromycin (GP) resistances (Fig. 2A), but no tetracycline and puromycin (TP) dual-resistant mycoplasmas were recovered (data not shown). ICEA transfer is therefore the main conjugative process observed between the PG2 mating partners used in these assays, since chromosomal exchange that would have conferred TP dual resistance was not detected regardless of the conditions (Fig. 1, mating A).

**Fig 3:**
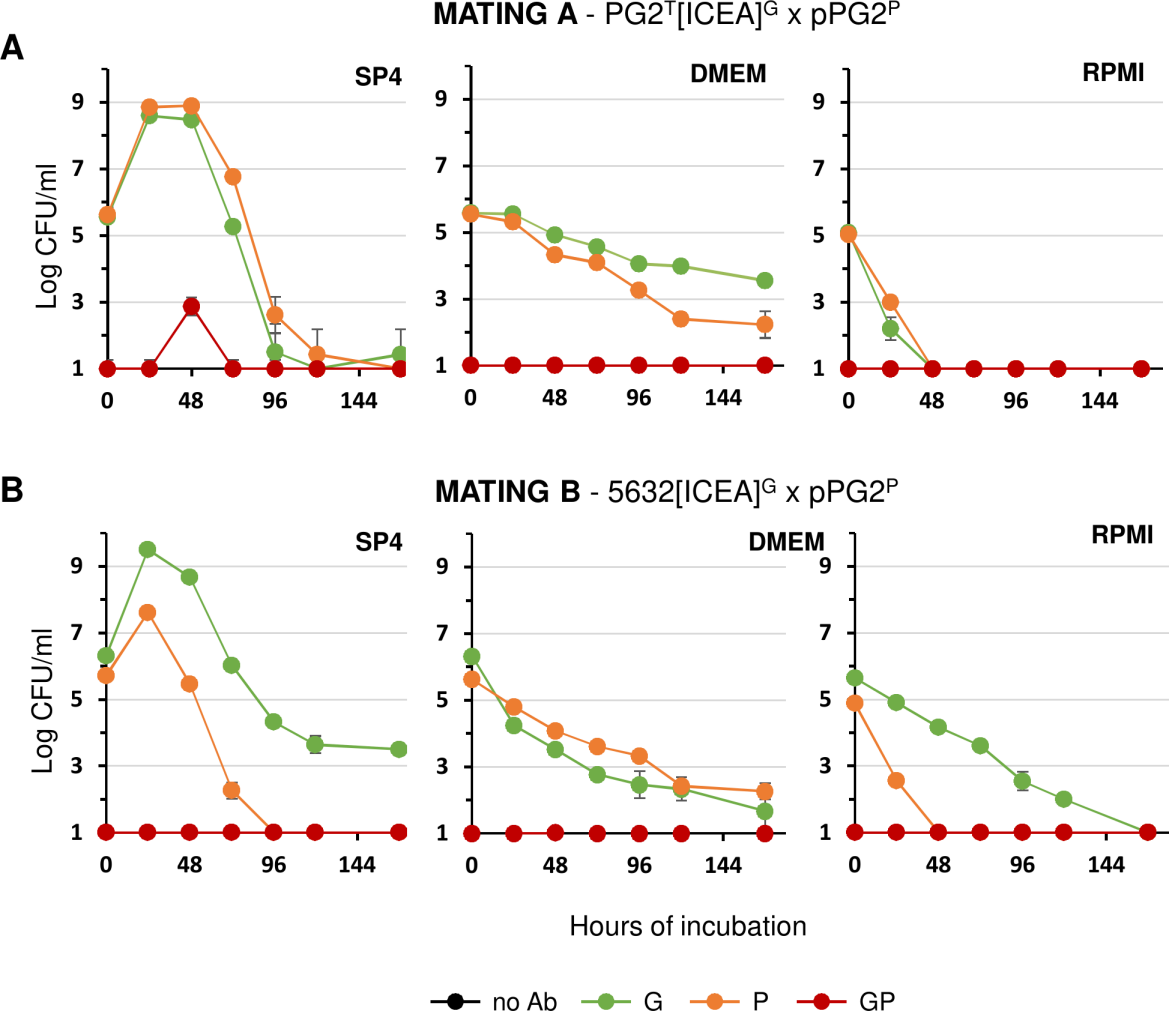
Mating experiments with *M. agalactiae* strain PG2 and 5632 in culture media. Variation of mycoplasma titers in SP4, DMEM, and RPMI media upon co-incubation of PG2^T^[ICEA]^G^ and pPG2^P^ (**A**), or 5632[ICEA]^G^ and pPG2^P^ (**B**). Mating partners (10^6^ CFU each) were co-incubated in 1 mL of medium. Antibiotics used for selection are color-coded (see Fig. 2). Data are the means of three independent assays. Standard deviations are indicated by error bars.

In each assay, the growth curve of the dual-resistant colonies mirrored that of the parents: in axenic conditions, GP dual resistance remained stable and low (ca. 10^3^ CFU/mL), while a remarkable increase in the number of dual-resistant colonies was observed upon co-incubation with epithelial cells, reaching up to 10^5^ CFU/mL at 72 h. Remarkably, when mating frequencies were calculated at 16 h, the ratio of dual-resistant to total CFUs was approximately 200-fold higher in cell culture compared to axenic conditions and remained nearly stable over time (Fig. 2B and Table 2). These data suggest that the conjugative properties of *M. agalactiae* may be influenced by environmental factors such as those provided by cell culture conditions. However, mating frequencies are also likely to be influenced by differences between the two mating procedures, such as the density of mating partners, the composition of the incubation medium, or even the centrifugation step. To address these issues, axenic mating experiments were performed following the conditions set for cell culture, using low titers of mating partners and no centrifugation step (Fig. 3A). These modified axenic conditions had a negative effect on conjugation since GP dual resistance could only be detected during the late stationary phase (48 h) following proliferation of the mating partners (Fig. 3A, SP4 medium). As expected, only sporadic or no conjugative activity was detected when the SP4 medium was replaced by Dulbecco’s modified Eagle’s medium (DMEM) (Fig. 3A), since mycoplasmas are unable to proliferate in cell culture media in the absence of host cells (24, 25). Finally, to determine whether the physical presence of host cells was the real cause of the observed effect on conjugation, as opposed to the chemical modification of the medium induced by eukaryotic cell growth, mating frequencies were determined by incubating the mating partners in conditioned cell culture medium (Fig. 2C). The conditioned medium used was filtered-sterile DMEM collected after pre-culture with the epithelial cells. After 24 h of incubation in conditioned DMEM, only a few dual-resistant colonies were selected, with no increase (DMEM conditioned for 24 h) or moderate increase (DMEM conditioned for 72 h) in mating frequency, which remained 150-fold lower than the mating frequency measured in the presence of epithelial cells (Table 2, mating A). Altogether, these results indicate that co-incubation with host cells is not only necessary for mycoplasma proliferation under cell culture conditions but also stimulates conjugation.

**TABLE 2:** Mating frequency of *M. agalactiae* and *M. bovis ex vivo*

Mating	ICE-positive partner^*b*^	ICE-negative partner	Mating frequency (×10^−6^)^*a*^		
			AXN	CELL	PCLS
A	PG2^T^[ICEA]^G^	pPG2^P^	2.6 ± 0.4	670 ± 180	66.9 ± 96
B	5632[ICEA]^G^	pPG2^P^	0.3 ± 0.1	68 ± 8.1*	3.9 ± 3.0
C	P3/CELL	pPG2^P^	ND	2.4 ± 1.1	ND
D	P3/AXN	pPG2^P^	ND	25 ± 4.1	ND
E	5632^T^	PG2^G^	0.3 ± 0.1	1.5 ± 0.7*	0
F	J228[ICEB]^G^	pRM16^P2^	0	7.2 ± 4.7	ND

^*a*^ Frequencies are given for 24 h of incubation under axenic (AXN), cell (CELL), and organotypic (PCLS) culture conditions. Asterisks denote values obtained at 48 h incubation when no mating progeny has been selected before. ND, not determined. The values are expressed as means ± standard deviations. The number of independent assays was 3 for each condition, except for mating C under CELL conditions, which was 9; mating B under PCLS conditions, which was 13; and mating A and E under PCLS conditions, which was 16.

^*b*^ P3/CELL and P3/AXN designate 5632[ICEA]^G^ populations generated upon three serial passages under cell culture and axenic conditions, respectively. For axenic passages, 1 mL SP4 was inoculated with 10 µL of the previous 5632[ICEA]^G^ population and further incubated at 37°C for 48 h. For cell culture passages, six-well plates at a density of 4 × 10^4^ epithelial cells/cm² in 3 mL DMEM were inoculated with 30 µL of the previous 5632[ICEA]^G^ population and further incubated at 37°C for 72 h.

Axenic and cell culture mating progenies were further characterized by mapping the chromosomal ICEA-integration sites. For each mating condition, 180 dual-resistant clones were isolated at 16 h. Individual clones were characterized by multiplex PCR amplification targeting the ICEA CDS22 and the chromosomal region surrounding the ICEA integration site in PG2^T^[ICEA]^G^ (Table S1). These amplifications (i) confirmed the presence of an ICEA in each clone and (ii) ruled out the acquisition of ICEA by chromosomal DNA exchange between mating partners (data not shown). Dual-resistant clones were then pooled, and their genomic DNA was extracted and sequenced using Illumina technology. Reads spanning DNA junctions between ICEA and the chromosome were mapped on the PG2 reference genome as described in Materials and Methods. Sequencing data revealed a broad distribution of ICEA-integration sites over the chromosome and identified a similar number of unique integration sites for axenic (67 sites) and cell culture (69 sites) conditions (Fig. 2D; Tables S2 and S3).

Although only 40% of ICEA integration sites were detected, the data are consistent with the previously documented absence of a specific chromosomal integration site for ICEA and further illustrate the high genetic heterogeneity resulting from ICEA transfer (8, 20). More importantly, the number and distribution of ICEA-integration sites in both mating progenies also rule out the hypothesis of the early expansion of a few highly fit, dual-resistant clones whose proliferation would have been responsible for the high dual-resistant titers detected under cell culture mating conditions.

Altogether, these results show that co-culture with host cells results in a higher frequency of ICEA transfer in *M. agalactiae* when compared to axenic conditions.

### *M. agalactiae* conjugation can be influenced by the fitness of the mating partners

To further investigate the conjugative properties of *M. agalactiae* under cell culture conditions, mating experiments were conducted with strain 5632, which is genetically and phenotypically distant from PG2 (27). This was performed by using the clone 5632[ICEA]^G^ as ICEA donor and pPG2^P^ as ICEA recipient (Fig. 1, mating B). The G-tagged ICEA in 5632[ICEA]^G^ is identical to PG2^T^[ICEA]^G^ (Table 1). Of note, the 5632 naturally contains three ICEA copies, and therefore, 5632[ICEA]^G^ possesses two additional untagged ICEA copies, both of which are functional and nearly identical to the G-tagged ICEA copy (15, 22). As a consequence, GP dual resistance only monitors the self-dissemination of a single ICEA copy from 5632[ICEA]^G^ to pPG2^P^, since the movements of the untagged ICEA copies cannot be monitored. Finally, in this experiment, chromosomal DNA exchanges could not be monitored. In previous studies, this event was shown to only occur from PG2 to 5632 due to differences in restriction-modification systems (15, 22). Here, chromosomal DNA transfer from pPG2^P^ to 5632[ICEA]^G^ would generate non-viable transconjugants due to the presence of a Hsd-5632 restriction recognition motif in the P resistance gene used as chromosomal tag in pPG2^P^ (30) (Table 1).

**Fig 4:**
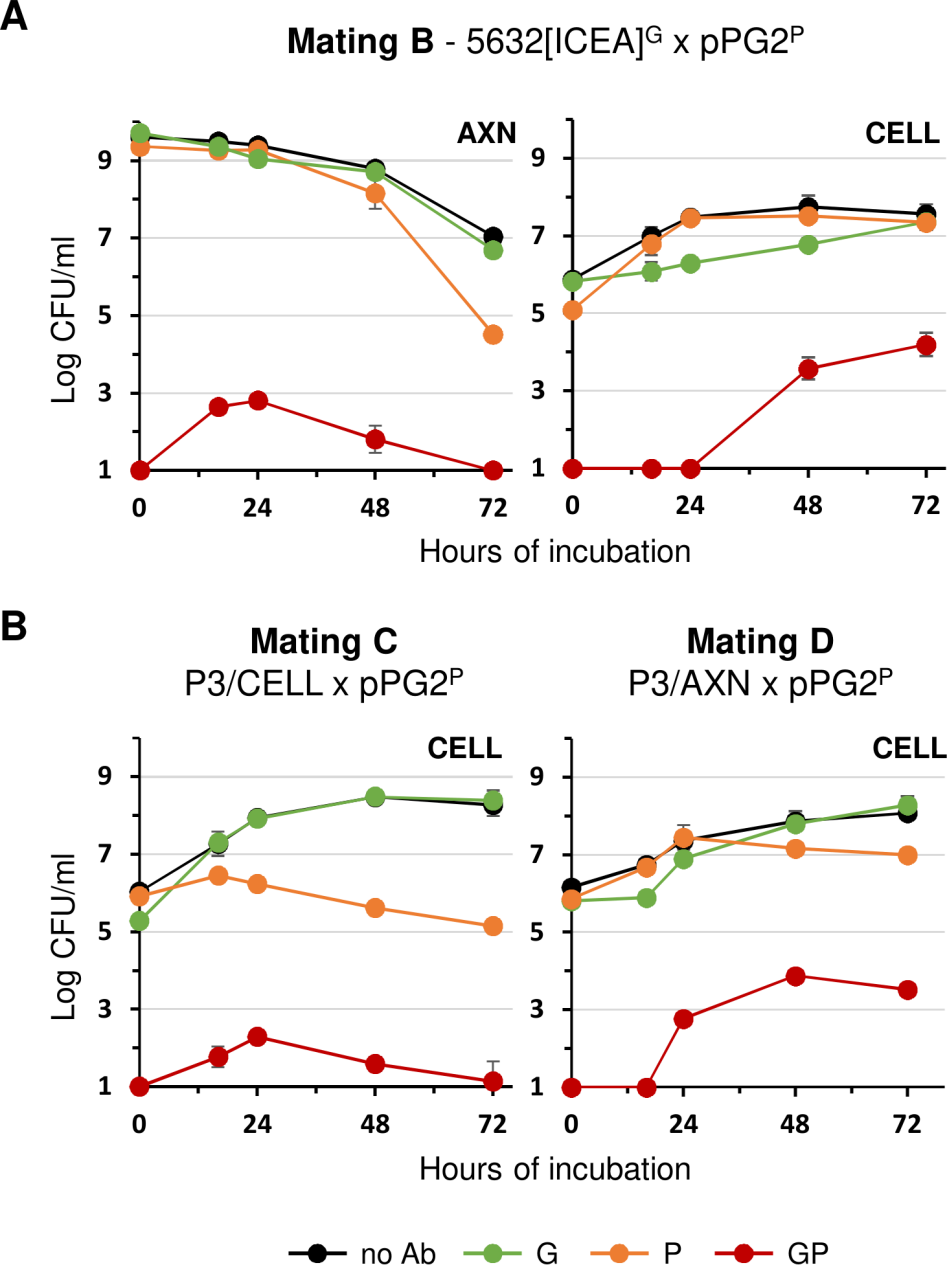
Mating experiments with *M. agalactiae* strain 5632 under axenic (AXN) and cell culture (CELL) conditions. (**A**) Variation of mycoplasma titers under AXN and CELL mating conditions with the ICEA-positive 5632 and the ICEA-negative PG2 mating partners (mating B in Fig. 1). (**B**) Conjugative properties of P3/AXN and P3/CELL in CELL mating conditions (mating C and D in Fig. 1). Antibiotics used for selection are color-coded (see Fig. 2). Data are the means of at least three independent assays, except for P3/AXN and P3/CELL, where data are means of three independent populations. Standard deviations are indicated by error bars.

Under axenic conditions, 5632[ICEA]^G^ was found to outcompete pPG2^P^ ICEA recipient cells, inducing a concomitant decrease in the selection of GP dual-resistant colonies (Fig. 4A). This is likely due to the previously documented fitness advantage of 5632 over PG2 under axenic conditions (17). Another notable difference with PG2^T^[ICEA]^G^ was the lack of dual-resistant colonies selected shortly after the centrifugation step (compare 0 h of incubation in Fig. 2A and 4A). This result suggests either a resistance of 5632[ICEA]^G^ to centrifugation-induced fusion with pPG2^P^ or a difference in ICEA activation between these two strains.

Despite the fitness advantage of 5632 over PG2 under axenic conditions (17), 5632[ICEA]^G^ showed delayed growth under cell culture conditions, reaching titers comparable to pPG2^P^ only after 72 h (Fig. 4A). Similarly, the detection of dual-resistant colonies was delayed by 48 h when compared to mating with PG2^T^[ICEA]^G^. As expected, no conjugative activity could be detected under axenic conditions conducted with cell culture settings, either in SP4 or DMEM medium (Fig. 3B). These results highlighted important differences in the conjugative properties of PG2^T^[ICEA]^G^ and 5632[ICEA]^G^ and identified growth competition between mating partners as an important factor affecting conjugation.

Further demonstration of how partner fitness can influence conjugation came from mating experiments with cell culture-propagated populations of 5632[ICEA]^G^ (Fig. 4B). Three populations (designated P3/CELL) were generated by three serial passages in cell culture, and three control populations (designated P3/AXN) were obtained upon further propagation in SP4 axenic medium. When compared to the parental 5632[ICEA]^G^, P3/CELL populations showed an improved ability to proliferate in cell culture conditions without any delay in growth and a capacity to outcompete pPG2^P^ (Fig. 4B, mating C). Consistently, dual-resistant colonies increased shortly after co-incubation of the two partners and then decreased in parallel with ICEA recipient cells. As expected, the behavior of the control population under cell culture conditions was similar to that of the parental 5632[ICEA]^G^, but with enhanced growth after 24 h of co-incubation (Fig. 4B, mating D).

In conclusion, *M. agalactiae* conjugation is influenced not only by the replicative environment but also by the relative fitness of each mating partner in that environment.

### Co-culture with host cells also promotes the self-dissemination of *M. bovis* ICEB

**Fig 5:**
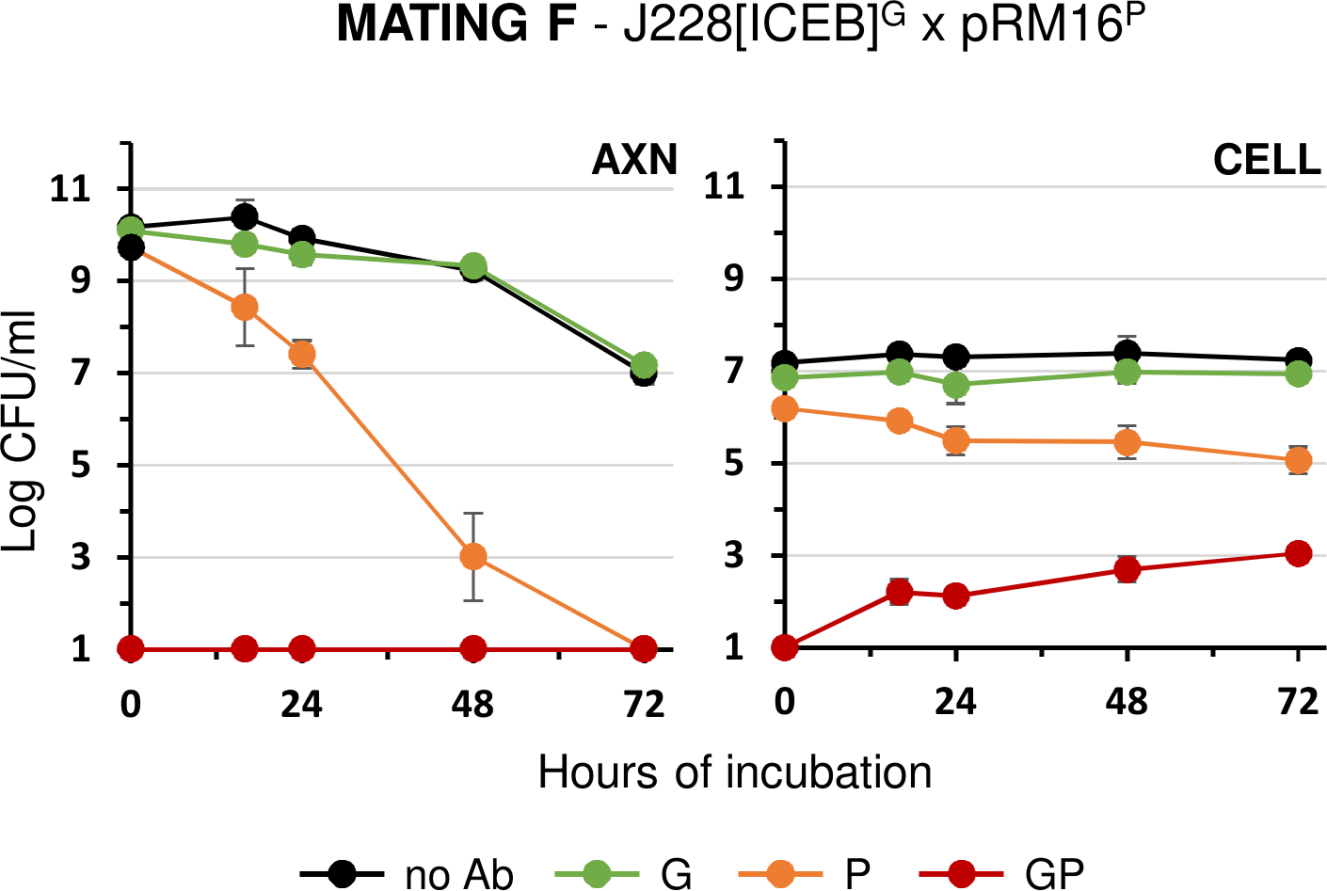
Mating experiments with *M. bovis* strain J228 under axenic (AXN) and cell culture (CELL) conditions. Variations in mycoplasma titers under AXN and CELL mating conditions with the ICEB-positive J228 and the ICEB-negative RM16 mating partners (mating F in Fig. 1). Antibiotics used for selection are color-coded (see Fig. 2). Data are means of at least three independent experiments. Standard deviations are indicated by error bars.

To further test the influence of host factors on mycoplasma conjugation, mating experiments were conducted with *M. bovis*, a ruminant mycoplasma species closely related to *M. agalactiae*. The clone J228[ICEB]^G^ was used as ICEB donor and a pool of five RM16^P^ clones (further designated pRM16^P^) as ICEB recipient (Table 1 and Fig. 1, mating F). Under axenic conditions, J228[ICEB]^G^ was found to outcompete pRM16^P^, and no dual-resistant progeny was detected (Fig. 5). Remarkably, co-incubation of these *M. bovis* partners under cell culture mating conditions led to progressive accumulation of dual-resistant colonies with titers reaching nearly 10^3^ CFU/mL, despite no obvious proliferation of the mating partners (Fig. 5). These data suggest that the effect of mycoplasma co-culture with host cells on conjugation is not limited to *M. agalactiae* and can also be observed with other ruminant mycoplasma species.

### Mycoplasma co-cultivation with bovine PCLS results in high-frequency ICEA transfer

**Fig 6:**
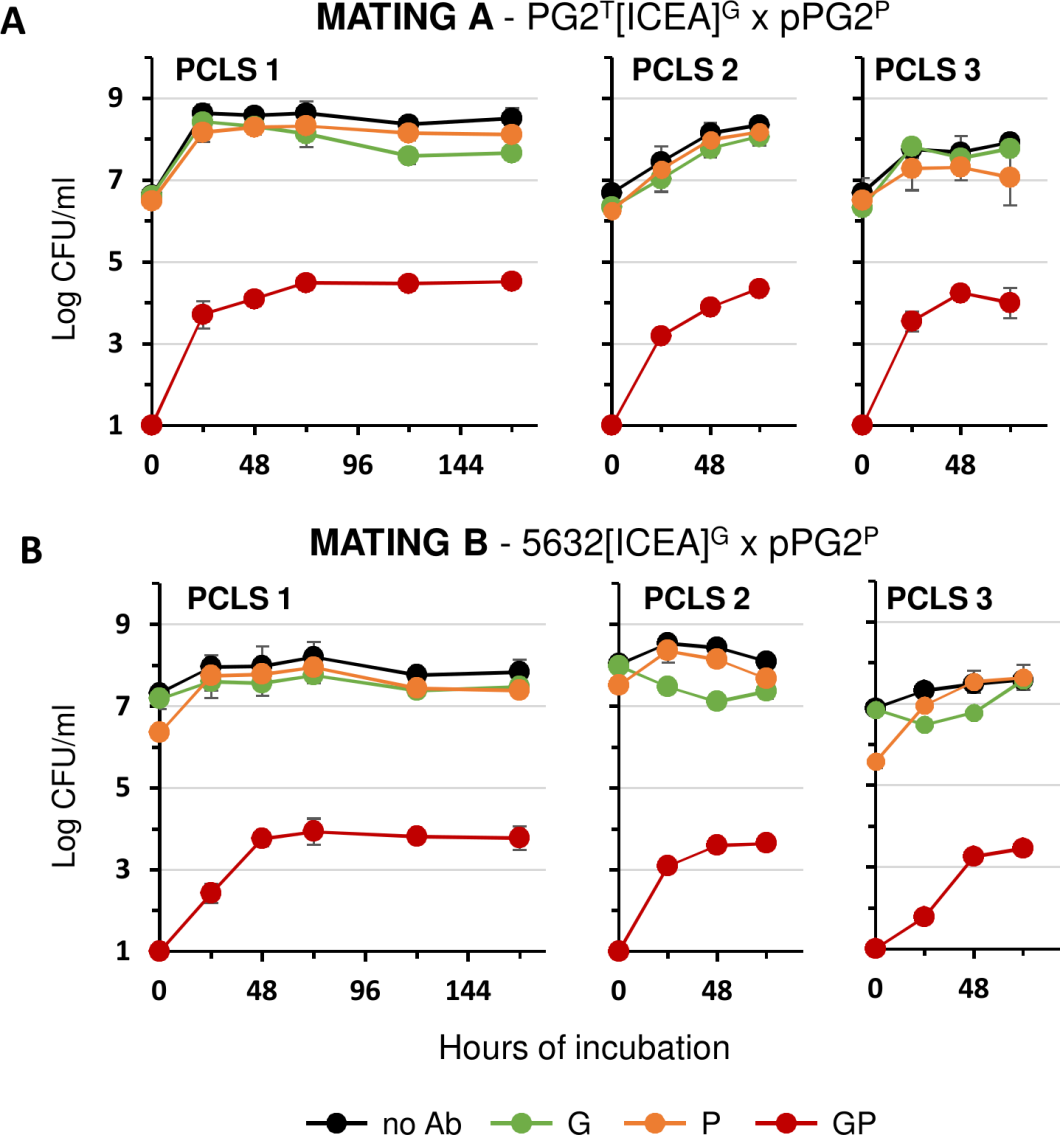
Mating experiments with *M. agalactiae* strain PG2 and 5632 under organotypic mating conditions. (**A**) Variation of mycoplasma titers under organotypic (PCLS) mating conditions with ICEA-positive and ICEA-negative PG2 mating partners (mating A in Fig. 1). PCLS were from three calf donors (PCLS 1 to 3). Data from PCLS calf donors 1, 2, and 3 are the means of six, four, and six independent assays, respectively. (**B**) Variation of mycoplasma titers under organotypic (PCLS) mating conditions with the ICEA-positive 5632 and the ICEA-negative PG2 mating partners (mating B in Fig. 1). Antibiotics used for the selection are indicated with a color code (see Fig. 1). Data from PCLS calf donors 1, 2, and 3 are the means of six, four, and three independent assays, respectively. Standard deviations are indicated by error bars.

Bovine PCLS were used to test ICEA transfer under organotypic culture conditions. PCLS were prepared from three calf lung donors and remained viable over an extended incubation period with up to 85% of the cells still viable on day 5 (see Materials and Methods). Mating experiments were conducted by using either PG2^T^[ICEA]^G^ or 5632[ICEA]^G^ as ICEA donor and pPG2^P^ as ICEA recipient (Fig. 1, mating A and B). As observed in cell culture, PG2^T^[ICEA]^G^ growth was stimulated by co-incubation with bovine PCLS (Fig. 6A). The behavior of 5632[ICEA]^G^ was dependent on the lung donor, showing either proliferation similar to PG2^T^[ICEA]^G^ with one PCLS (Fig. 6B, PCLS 1) and delayed growth with the other two (Fig. 6B, PCLS 2 and 3). Mycoplasmas remained viable even after a prolonged period of co-incubation, with titers ranging from 10^7^ to 10^8^ CFU/mL. Remarkably, GP dual-resistant colonies were observed within 24 h of co-incubation, not only with PG2^T^[ICEA]^G^ but also with 5632[ICEA]^G^. Maximum titers of dual-resistant colonies were observed after 48 to 72 h of co-incubation and remained nearly stable even after prolonged co-incubation, likely due to limited competition between mating partners (Fig. 6A and B). As expected, mycoplasmas were unable to proliferate upon incubation in RPMI medium without PCLS, and only sporadic conjugative activity was detected (Fig. 3A and B). These results indicate that ICEA transfer can occur at high frequency under organotypic culture conditions.

### Mosaic mycoplasma genomes are generated under cell and organotypic mating conditions

**Fig 7:**
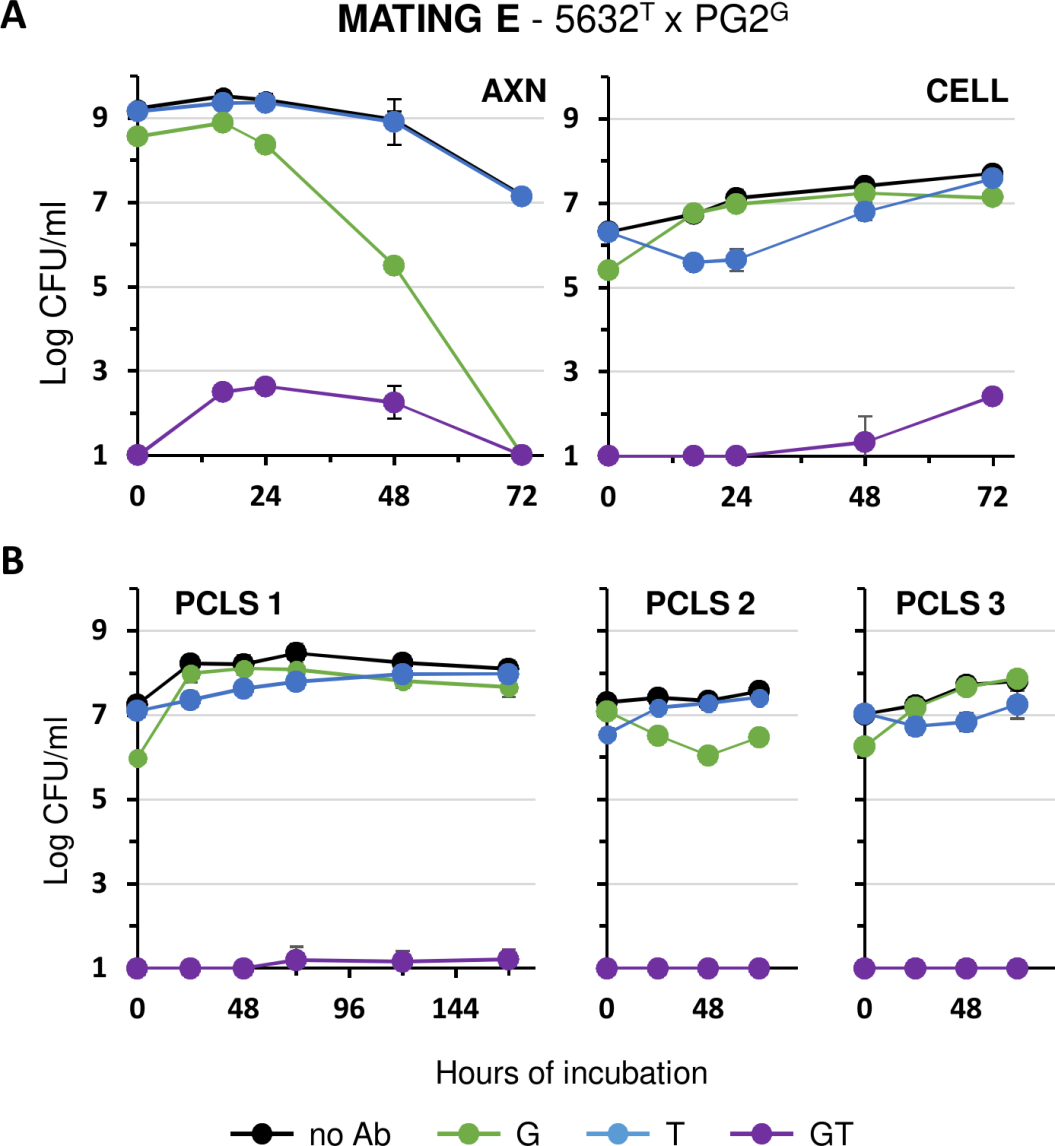
Chromosomal transfer in *M. agalactiae* under axenic (AXN), cell culture (CELL), and organotypic culture conditions. Variation of mycoplasma titers under (A) AXN, CELL, and (B) organotypic (PCLS) mating conditions with the ICEA-positive 5632 and the ICEA-negative PG2 mating partners (mating E in Fig. 1). PCLS were from three calf donors (PCLS 1 to 3). Antibiotics used for selection are color-coded (blue for T, green for G, and purple for T and G). Mycoplasma titers without Ab selection are shown in black. Data are the means of three independent assays for axenic and cell culture mating conditions. Data from PCLS 1, 2, and 3 are the means of six, four, and three independent assays, respectively. Standard deviations are indicated by error bars.

The high frequency of GP dual-resistance generated upon mating with 5632[ICEA]^G^ and pPG2^P^ under cell and organotypic culture conditions led us to evaluate the rate of chromosomal transfer between these two mating partners. This was done by using a 5632^T^ ICEA-positive partner and a PG2^G^ ICEA-negative partner, each labeled by a specific Ab-tag inserted in their genome (Table 1). Since the 5632^T^ ICEA-positive partner has no tagged ICEA (Fig. 1, mating E), TG dual-resistant colonies will only result from the unidirectional transfer of chromosomal DNA from PG2^G^ to 5632^T^ and the creation of mosaic genomes. As expected, the growth of 5632^T^ and PG2^G^ was very similar to that described for 5632[ICEA]^G^ and pPG2^P^ under both axenic and cell culture mating conditions (Fig. 7A). However, only a limited number of dual-resistant mosaic genomes could be selected in cell culture when compared to axenic mating conditions. This situation was even more dramatic upon co-incubation with bovine PCLS, with only a few or no dual-resistant mosaic genomes detected (Fig. 7B). This suggests either (i) a particular feature of the partners used in mating E, (ii) a lower frequency of MCT when compared to ICEA transfer, or (iii) the formation of mosaic genomes with limited survival under cell and organotypic culture conditions. However, further studies are needed to fully understand the particular growth and mating properties of strain 5632.

### Nucleotide stress may stimulate ICEA self-dissemination

**Fig 8:**
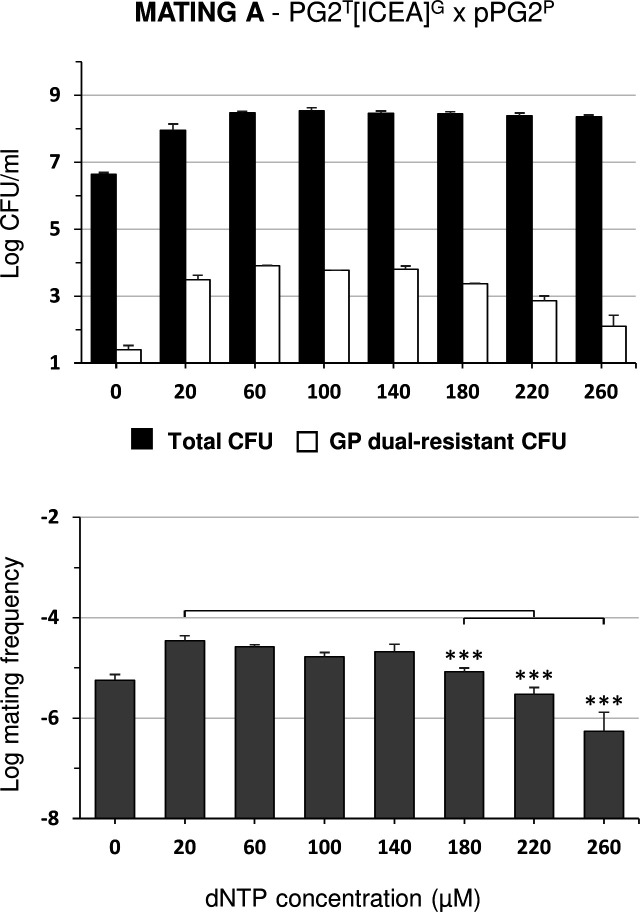
Influence of dNTPs on mating properties of *M. agalactiae* in cell culture medium. Mycoplasma titers and mating frequencies upon 24 h co-incubation of ICEA-positive and ICEA-negative PG2 mating partners in DMEM medium supplemented with increasing concentrations of dNTPs. No eukaryotic cells were added to the culture medium. The black and white bars show total mycoplasma titers and dual-resistant progeny selected with a combination of G and P, respectively. Data are the means of at least three independent assays. Standard deviations are indicated by error bars. The *P-*values were determined using two-tailed independent sample *t*-tests and comparing the mating frequency at a given dNTPs concentration to that at 20 µM dNTPs, which is the lower dNTPs concentration to achieve maximum mycoplasma titers without antibiotic selection (***, *P* < 0.001).

Due to their limited metabolic capacity, mycoplasmas are dependent on their hosts for many nutrients. Under cell culture conditions, nutrients that are essential for *M. agalactiae* and *M. bovis* are provided by eukaryotic cells (24, 25, 29). Interestingly, supplementation of cell culture media with DNA can overcome this limitation and stimulate mycoplasma growth even in the absence of eukaryotic cells (25). This prompted us to study the influence of nucleotides on ICEA transfer. Mating experiments were conducted in cell culture medium supplemented with dNTPs but without eukaryotic cells. PG2^T^[ICEA]^G^ and pPG2^P^ mating partners were inoculated at low density (10^6^ CFU/mL) and analyzed by titration after 24 h of incubation with increasing concentrations of dNTPs (Fig. 8). The addition of dNTPs was associated with a 10-fold increase in mycoplasma titer and the selection of dual-resistant colonies. Remarkably, elevated concentrations of dNTPs (ranging from 180 to 260 µM) resulted in a decrease in mating progeny with no discernible effect on mycoplasma growth, a situation that negatively affected mating frequencies. Further studies are needed to fully understand the influence of dNTP concentration on conjugation, but these results point to nucleotide stress as a possible nutritional factor capable of stimulating ICEA transfer in mycoplasmas.

## DISCUSSION

In addition to the control exerted by mobile genetic elements on their own dissemination, it is becoming clear that HGT among host-associated bacteria can be modulated by *in vivo* and in-host environmental conditions (1, 31–33). By using different *ex vivo* infection models, our study shows that eukaryotic cells can considerably impact the mating frequency of ruminant *Mycoplasma* spp.

### Mycoplasma ICE transfer *ex vivo*

Mating experiments with the prototype PG2 strain of *M. agalactiae* revealed elevated rates of ICEA transfer under cell culture and organotypic conditions, with a mating frequency reaching up to 7 × 10^−4^ dual-resistant colonies per total CFUs at 24 h. This value was 200-fold higher than those measured using the established axenic method for conjugation (20, 23). This difference was not the outcome of the outgrowth of a few high-fitted transconjugants upon co-incubation with host cells, since the distribution of chromosomal ICEA insertion sites was comparable in both conditions. However, ICEA transmission rates in cell culture were likely overestimated at longer incubation times due to repeated conjugation events and proliferating progeny. Without detectable MCT events, ICEA transfer was the major conjugative process stimulated by co-incubation with eukaryotic host cells.

Co-incubation with eukaryotic cells was able to stimulate conjugation not only in the prototype *M. agalactiae* strain but also when 5632 was used as ICEA donor. However, 5632 showed reduced conjugative activity under all conditions tested. This difference is consistent with previous studies conducted under axenic mating conditions, which reported mating frequencies of 1 to 8 × 10^−6^ and 5 to 10 × 10^−8^ for PG2 and 5632, respectively (15, 20, 22). Factors influencing ICE transfer from 5632 to PG2 include (i) the number of co-resident chromosomal ICEA copies in the donor cells since ICEA movements can only be monitored for the copy that contains an Ab-tag (22) and (ii) the relative fitness of each partner that can influence the ratio of donor and recipient cells.

Finally, co-incubation with eukaryotic cells also stimulated conjugation in *M. bovis*, providing the first report of ICEB transfer within this species. Indeed, no transfer had been detected between two *M. bovis* partners under the axenic conditions previously developed for ruminant mycoplasmas (23). Cell culture mating conditions thus provide a valuable model to study conjugation in this economically important pathogenic species.

### Mycoplasma chromosomal transfer *ex vivo*

Mycoplasma ICEs were found to play a central role in MCT, which requires a functional ICE in at least one of the mating partners (8, 15, 20). Both MCT and ICE transfers were previously documented as occurring with the same frequency in axenic conditions (15). Under cell and organotypic culture conditions and in contrast to ICE transfer, MCT was only sporadically observed when 5632 was used. Further studies are needed to fully understand this observation, but these two cellular environments may have a negative impact on the viability of the mosaic genomes resulting from MCT. Another hypothesis is suggested by our recent study that identified mycoplasma restriction-modification (RM systems as key in controlling chromosomal transfer (30). Indeed, RM systems may influence the polarity of DNA transfers by fragmenting the unmethylated chromosome of one partner, thereby facilitating its incorporation into the methylated chromosome of the second partner. Consequently, MCT may be influenced by the composition of the RM systems in each partner and by changes in their DNA methylation profile. This hypothesis is consistent with the absence of chromosomal exchanges between two partners having the same chromosomal background, such as mating experiments with two PG2 partners that failed to produce any MCT progeny under all the conditions tested. Whether adaptation of 5632 to cell and organotypic culture conditions induces changes in DNA methylation is unknown, but it is increasingly recognized that epigenetic modifications in bacteria can respond to the environment and modulate cell cycle, gene expression, and virulence (34, 35). In the human pathogen *Mycoplasma pneumoniae*, the methylation status is subject to variation as a function of the growth phase (36). Further studies are needed to characterize the epigenetic features associated with 5632 grown in the presence of eukaryotic cells and to determine their potential impact on MCT.

### Nucleotide stress as a potential trigger of mycoplasma conjugation

Nucleotide metabolism is emerging as critical to *M. bovis* survival and virulence (25, 26, 37, 38) and may also be key to *M. agalactiae* interaction with host cells and HGT. Indeed, dNTPs were found (i) to overcome the dependence of *M. agalactiae* on eukaryotic cells for proliferation in cell culture and (ii) to inhibit bacterial conjugation at high concentrations. This suggests that mycoplasma conjugation under cell culture conditions may be stimulated by a nutritional stress generated by fluctuations in nucleotide levels. Further studies are needed to elucidate the impact of nucleotide stress on the physiology of mycoplasma cells. However, it is known that starvation can lead to DNA damage and the induction of the SOS response in bacteria, a physiological condition known to trigger the activation of several ICEs in a RecA-dependent manner (5). Nevertheless, DNA repair mechanisms in mycoplasmas are still poorly understood, and no SOS response has been documented so far except for an SOS-like response in *Mycoplasma gallisepticum* (39).

Finally, while the dependence of *M. agalactiae* on eukaryotic cells can be overcome by dNTP supplementation, the presence of eukaryotic cells was needed to reach maximal conjugation rates. Indeed, mating frequencies induced by dNTP supplementation remain approximately 10-fold lower than those observed under standard cell culture conditions (compare mating frequencies at 24 h in Fig. 2B and 8). This suggests that multiple factors are likely involved, such as cell surface adhesion, which may facilitate the contact needed for mycoplasma conjugation to occur.

In conclusion, this study illustrates how eukaryotic host factors impact and promote conjugation in mycoplasmas, raising the possibility that *in vivo* HGT events may have been underestimated. This may be important when considering the growing number of pathogenic mycoplasma species harboring conjugative elements (21, 40–44). Finally, the *ex vivo* models developed here will be valuable for further deciphering the mechanisms and characteristics underlying mycoplasma conjugation during interactions with host factors.

## MATERIALS AND METHODS

### Mycoplasmas and culture conditions

Ab-tagged *M. agalactiae* and *M. bovis* strains used in this study are described in Table 1. *M. agalactiae* strains PG2 and 5632 (19, 27) differ in their ICEA content, with strain 5632 having three functional, almost identical ICEA copies and strain PG2 containing only vestigial forms (14, 16). Similarly, *M. bovis* strains RM16 and J228 (25, 28) differ in their ICEB content, with strain J228 having two chromosomal ICEB-2 copies and strain RM16 containing no ICEB (18).

Stock cultures were produced by growing mycoplasmas at 37°C in SP4 medium (45) supplemented with 100 µg/mL ampicillin (Sigma-Aldrich) and stored at −80°C. When needed, gentamicin (50 µg/mL, Gibco), puromycin (10 µg/mL, Thermo Fisher), and tetracycline (2 µg/mL, Sigma-Aldrich), alone or in combination, were added to the medium. Spontaneous resistance in stock cultures was tested by plating on selective media. Since mycoplasma growth cannot be monitored by optical density, mycoplasma titers were determined based on colony counts on solid SP4 media after 4 to 7 days of incubation at 37°C using a binocular stereoscopic microscope (24). The detection limit for mycoplasma titration was 100 CFU/mL, except for the detection of mating progeny, which was 10 CFU/mL.

### Genetic tagging of mycoplasmas with antibiotic markers

Selective antibiotic markers were introduced randomly in the mycoplasma genome by transforming mycoplasma cells with plasmid pMT85, which carries a G resistance mini-transposon (46), or its derivatives, pMT85-Tet, pMT85-Pur and pMT85-Pur2, in which the G resistance was replaced by a T or a P resistance (20, 24). Plasmid pMT85-Pur2 encodes a codon-optimized version of the puromycin resistance *pac* gene and a mutation at the Hsd-5632 recognition motif 5′-A^m6^YC(N)5KTR-3′ (30). J228[ICEB]^G^ was selected from a library of 300 mutants generated by random transposon mutagenesis and identified by its capacity to generate dual-resistant progeny upon mating with pRM16^P^ under cell culture conditions (see below). Whole-genome sequencing of PG2^T^[ICEA]^G^ identified the integration site of the G-tagged ICEA at PG2 genomic position 703341 (GenBank accession number CU179680.1) corresponding to the N-terminal region of a hypothetical protein with unknown function encoded by MAG6030. The PG2 chromosomal T-tag is located 83 nucleotides upstream of MAG1620 (genomic position 188668), which encodes a hypothetical protein.

### Epithelial cells and organotypic cultures

The T-antigen immortalized goat milk epithelial cell (TiGMEC) line was kindly provided by C. Leroux (UMR 754 INRAE; Université Lyon 1, Lyon, France) (29, 47). Cells were grown in DMEM (high glucose, sodium pyruvate, and GlutaMAX-I; Gibco) supplemented with non-essential amino acids (Gibco) and 10% heat-inactivated fetal bovine serum (FBS, Gibco) (24, 29). The mycoplasma-free status of the cell line was tested by using a genus-specific PCR (48).

The precision bovine lung slices (PCLS) used in this study were obtained from another study (49). Briefly, lung tissues were collected from 3- to 6-week-old male calves. Lung donors that were PCR positive for respiratory pathogens were excluded (49). PCLS of 100 µm thickness were prepared from 8 mm punch biopsies of cranial and accessorial lobes using the Krumdieck MD6000 tissue slicer (Alabama Research & Development) as described (49). PCLSs in 24-well plates were washed with RPMI medium (Gibco) supplemented with 10% FBS and 1% penicillin-streptomycin (10,000 U/mL penicillin and 10 mg/mL streptomycin, Pan Biotech). After overnight recovery at 37°C with 5% CO_2_ and two additional washes in RPMI, mating experiments were performed in RPMI supplemented with 10% FBS, amphotericin B (2.5 µg/mL, Sigma-Aldrich), and ampicillin (0.3 mg/mL, Sigma-Aldrich). PCLS viability data were published in reference 49.

### Mycoplasma mating experiments

Mating experiments with Ab-tagged mycoplasmas were conducted under axenic, cell, and organotypic culture conditions. Axenic mating conditions were as previously described (20, 23). Briefly, cultures of donor and recipient mycoplasmas were mixed in a 1:1 CFU:CFU ratio, centrifuged for 5 min at 8,000 × *g* at room temperature, resuspended in 1 mL of SP4 medium, and further incubated at 37°C. At the end of the incubation period, mycoplasmas were plated onto SP4 solid media supplemented with the appropriate antibiotics for selecting either the parental populations or the transconjugant progeny or without antibiotics for the total cell count. Cell culture mating experiments were performed by inoculating donor and recipient mycoplasmas to epithelial cell cultures. TiGMEC cells were seeded in six-well plates (Falcon) at a density of 4 × 10^4^ cells/cm² and inoculated at a mycoplasma:cell ratio of ca. 5 CFU/cell (10^6^ CFU/mL). Organotypic culture mating experiments were performed by inoculating ca. 10^6^ CFU of each mycoplasma partner to individual PCLS in RPMI medium. At different times post-inoculation, mycoplasma titers were determined by CFU titration on selective SP4 solid media following three freeze-thaw cycles of cell and organotypic cultures. Mycoplasma cell viability is not affected by limited freeze-thaw cycles due to the cryoprotective effect of the serum. Of note, mating experiments in cell culture media alone (DMEM or RPMI) without cells were not referred to as axenic conditions because they do not support mycoplasma growth. For mating A, B, E, and F (Table 2), 10 dual-resistant colonies were controlled by PCR amplifications using primers targeting the resistance genes used for their selection (Table S3). For mating A, B, and E, these dual-resistant colonies were further tested with PCR amplifications using primers targeting CDS22 in ICEA (Table S3).

### Mycoplasma whole-genome sequencing and bioinformatic mapping of ICE-integration sites

Mycoplasma genomic DNA was purified by phenol/chloroform extraction and ethanol precipitation (50). Whole-genome sequencing was performed using Illumina sequencing technology (NovaSeq PE 150). Raw sequencing data were generated at Eurofins Genomics (Germany). Bioinformatics analyses were conducted using the GenoToul Bioinformatics facility in Toulouse, France (http://bioinfo.genotoul.fr/).

To map ICE-integration sites from large pools of transconjugants, Illumina raw sequencing data, after quality control and sequencing adapter trimming, were filtered to select direct and indirect reads spanning DNA junctions between ICEA and the mycoplasma chromosome. This was done by selecting reads containing nucleotide sequences GGAACTGATATAAGAAAGTG or reverse complement CACTTTCTTATATCAGTTCC corresponding to 5′-ICEA ends (hereafter referred to as CDS1 reads) and nucleotide sequences CCCACTTAATACTTTCATTC or reverse complement GAATGAAAGTATTAAGTGGG corresponding to 3′-ICEA ends (hereafter referred to as CDS22 reads). Selected reads were mapped on the PG2 reference genome (National Center for Biotechnology Information accession number CU179680.1) with BWA_MEM (51). BAM files corresponding to aligned reads were processed with BEDTools Intersect v.2.29.1 (52) to identify overlapping CDS1 and CDS22 reads. The intersection files, generated by BEDTools Intersect v.2.29.1, were imported into Artemis v.16.0.0 (53), where each CDS1/CDS22 intersection was converted into a feature on the PG2 reference genome. Features were created based on the number of reads at each potential ICEA insertion site, with a minimum number of four reads for each ICEA extremity. This cutoff was selected after manual reconstitution and inspection of each feature to identify false ICEA insertion sites. Selected ICEA integration sites were further examined to confirm the presence of an eight-nucleotide direct repeat on the overlap between CDS1 and CDS22 reads, which is a characteristic signature of ICEA integration events.

## Data Availability

The data sets for this study can be found at https://www.ebi.ac.uk/ (accession numbers ERR14088891, ERR14088864, and ERR14088863).

## SUPPLEMENTAL MATERIAL

Fig. S1 aem.00868-25-s0001.tif PCR characterization of dual-resistant colonies.

Supplemental tables aem.00868-25-s0002.docx Tables S1 to S3.

ASM does not own the copyrights to Supplemental Material that may be linked to, or accessed through, an article. The authors have granted ASM a non-exclusive, world-wide license to publish the Supplemental Material files. Please contact the corresponding author directly for reuse.
